# A Clinical Study of the Small Granular Cells of the Blood

**Published:** 1882-09

**Authors:** James T. R. Davison

**Affiliations:** Edinburgh; Late Resident Physician to the Royal Infirmary, Edinburgh; House Surgeon to the Royal Southern Hospital, Liverpool


A CLINICAL STUDY OF THE SMALL GRANULAR CELLS OF THE BLOOD. By JAMES T. R. DAVISON, M. D. Edinburgh. Late Resident Physician to the Royal Infirmary, Edinburgh; House Surgeon to the Royal Southern Hospital, Liverpool.

Blood contains, in addition to the red and white corpuscles, a number of granular colorless cells, which present varied forms and sizes, and are generally very much smaller than the red corpuscles when examined in the ordinary way. They have a great tendency to adhere to the cover-glass or slide; they occur singly or in groups. Some time after the blood has been drawn from the finger these cells become clearer; if present in groups the individuality of the cells is lost, and the group becomes a single granular mass. In either case fibrin or fibrin-like processes protrude from their surface. These cells have been known for some time to Professor Norris, who has traced them in their development, and states them to be undeveloped red corpuscles. Bizzozero has lately studied them in the living capillary. In healthy blood they are present in extremely small numbers, but are very abundant in certain pathological states. I have examined these cells in 150 specimens of pathological blood, noting in each case the temperature of the patient from whom the blood was drawn, whether anæmic or not ( in the last 100 cases, if anæmic, I noted roughly, the degree anæmia present), and lastly the nature and duration of the disease.

My observations lead me to believe that the small granular cells of the blood are present in excess under two conditions—first, as the result of their excessive formation brought about by certain states of the system demanding it; and secondly, as the result of diminished power in the process of charging them with hæmoglobin, whereby many of them remain in their undeveloped state, and do not become fully formed red cells.

In the inflammatory exudation produced by a blister, Dr. Burdon-Sanderson years ago found granular corpuscles which degenerated into fibrin. These corpuscles must be the cells under consideration at present, for these cells are constantly seen to change into fibrin, while leucocytes never undergo this change. Here, then, we have the important fact that in inflammatory serous exudation, such we have in pleurisy, rheumatic fever, etc., the small granular corpuscles of the blood migrate from the blood-vessels. Dr. Burdon-Sanderson in his first Lumleian lecture this year described ordinary inflammation of the mesentery, and after referring to the migration of leucocytes he states that the surface of the mesentery becomes covered by a coagulable fluid. This coagulum, when examined under the microscope, is seen to consist of fibrin which Dr. Burdon-Sanderson ascribes to the degeneration of leucocytes. Now, if a drop of blood be examined under the micrôscope the leucocytes are seen to remain intact for a long time, and never undergo the same changes as the small granular cells—that is, they never degenerate into fibrin. Moreover, Professor Norris has found that the fibrin of coagulation is due to changes in the undeveloped red cells. We must come to the conclusion, then, that the coagulable fluid on the surface of the mesentery was full of undeveloped red cells, which had degenerated into fibrin—that is, that in ordinary inflammation, in addition to the migration of leucocytes. there is a migration of the small granular corpuscles. We have good grounds, then, for believing that not only in serous inflammatory exudation, but also in purulent exudation, there is a migration of the undeveloped red corpuscles. If then, in local inflammation the blood is drained of its small granular corpuscles, nature must in some reflex manner stimulate the formative organs of these cells to increased action, in order to meet the demand made upon the blood. Hence in local inflammations we would expect to find an increase of these cells in the blood. I will not refer in these notes to the part played by leucocytes in the blood under these conditions. Such an increase we find in inflammation. In acute abscesses the blood is loaded with undeveloped red cells. In a case of acute periostitis with a large amount of pus these cells were found abundant in the blood. In rheumatic fever and subacute rheumatism they are again present in abundance. In these last conditions we have serous inflammation in the joints, making a great demand upon the blood. During the fever of pneumonia I did not find the increase so marked as in rheumatic fever. Possibly the inflammatory exudation thrown out in pneumonia does not contain many small granular cells. That high temperature *per se* is not the cause of the increase of these cells was proved in a case of typhoid fever, where, after a fortnight of high temperature, there was scarcely any increase of these cells; also in another case of typhoid, with a temperature of 103.4°, and of five weeks' duration, the increase was very small. In typhoid there is no drain from the blood, at least no loss of the solid element of the blood, and therefore no demand is made for an increase in the production of these elements. In great loss of blood from hæmorrhage there is a great loss of the red corpuscles, and on the same principle we would expect after a time to find an increase of the undeveloped red cells, in order that they should replace the lost red cells. If after excessive hæmorrhage we examine the blood, we actually find a great increase in the number of the small granular cells. Another source of drain of the solid elements from the blood is found in chronic discharges of pus. In cases of spinal caries with discharge of pus, in cases of necrosis, in those where any large wound is constantly secreting pus—in fact, in all cases where there is chronic pus formation, the blood is full of undeveloped red cells. I shall show further on that a drain of the liquid element of the blood, however great, does not affect the number of these cells in the blood.

I will next consider the increase of the small granular cells, in relation to anæmia. There appear to be two factors in the production of the anæmic state, both of which may be present together, but not necessarily. One factor is a lowering of the power by means of which the small granular corpuscles are charged with hæmoglobin, and changed therefore into fully developed red cells; the other is a lowering of the formative power, which creates the small granular corpuscles, and therefore which creates the fully formed red cells. In the one case the red cells, developed and undeveloped, are present in normal quantity, but the latter are in relative excess. Such a condition would obtain in chlorotic anæmia, where there is no loss of substance in the body, and therefore we would infer no loss of substance in the cellular element of the blood, but where an impaired vitality retards the full development of the red cells. Such a condition would obtain, also, in the convalescence from acute diseases. I have found, as a matter of fact, abundance of small granular corpuscles in the blood of chlorotic patients, and in that of those convalescing from rheumatic fever and pneumonia.

In the second kind of anæmia the predominant factor is the lowering of the formative energy. As a typical example we have the anæmia present in the malignant dyscrasia. Here there is a general loss of substance, not from any high temperature, as this may be altogether absent, but apparently from a general impairment of formative power. If thus the cells of the different organs suffer in their numerical formation, we may naturally suppose that the red cells of the blood are also placed in the same condition. This appears to me the only explanation which can account for the small increase of the undeveloped red cells which I have observed in cases of great anæmia and emaciation due to malignant growths. It appears to me that the red cells are diminished in number, so that the energy which charges the undeveloped ones with hæmoglobin has a smaller area over which to exert its strength, and thus although this energy too must necessarily be greatly impaired, and as a consequence some of the small granular cells must remain undeveloped, still the impaired energy has less work to do, there being fewer small granular cells to develop, and hence it is that in such conditions we find in the blood but a small increase in the number of the small granular cells. I believe that the same explanation must account for the small increase of the cells which I observed in a case of chronic dysentery with great anæmia and emaciation. The constant albuminoid discharge from the intestines appears to exert the same influence on the formative energy as in the case of the malignant dyscrasia. Lastly, the small increase observed in the cases of chronic Bright's disease must be explained in the same way. In the cases of chronic Bright's diesease and dysentery the blood is drained of its liquid element, and the increase of the undeveloped red cells is small; in those cases where the drain consisted of solid elements the increase was marked. It must be borne in mind that the two conditions under which an increase of the small granular cells is present may exist together in the same case. For example, in phthisis, where these conditions are typically present, these cells are very abundant. In phthisis we have cavities secreting pus, hence there is a reflex call for an increase in the formation of the granular cells. But in phthisis there is also a general impairment of vitality, therefore the power to charge these cells with hæmoglobin is weakened, and hence the very large number of undeveloped red cells seen in the blood of phthisical patients. Again, in cancer the disease of these cells, as explained above, is small, but let the cancer bleed or let it constantly discharge, and matters will be different. We now have a drain of the solid elements of the blood, with the accompanying increased reflex formation, and so we find, in bleeding cancers or in those from which there is a discharge, an abundance of small granular corpuscles in the blood.

In conclusion, I have purposely avoided in this paper any reference to the behavior of leucocytes in the blood under the conditions in which the other cells have been considered. The increase of leucocytes in the blood under certain conditions is important, and the relation of their increase to that of the small granular cells in the same blood may one day prove to be of great diagnostic value. There is some ground for believing that, in certain classes of inflammations, the leucocytes are increased in greater relative proportion to the small granular cell, and *vice versa.* By a knowledge of the conditions under which either increase takes place, we may, perhaps, be able, by an examination of the blood, to determine whether an ordinary pleurisy has become an empyema, and to arrive at other diagnosis. A clinical study of the blood in all cases is, therefore, of the highest importance.—*London Lancet.*

